# Micro-Behavioral Accidental Click Detection System for Preventing Slip-Based Human Error

**DOI:** 10.3390/s21248209

**Published:** 2021-12-08

**Authors:** Abdulaziz Almehmadi

**Affiliations:** SNCS Research Center, Department of IT, Faculty of Computing and IT, University of Tabuk, Tabuk 71491, Saudi Arabia; aalmehmadi@ut.edu.sa

**Keywords:** human error detection, system design, accidental click detection, smartphone sensors, security, artificial intelligence

## Abstract

Accidentally clicking on a link is a type of human error known as a slip in which a user unintentionally performs an unintended task. The risk magnitude is the probability of occurrences of such error with a possible substantial effect to which even experienced individuals are susceptible. Phishing attacks take advantage of slip-based human error by attacking psychological aspects of the users that lead to unintentionally clicking on phishing links. Such actions may lead to installing tracking software, downloading malware or viruses, or stealing private, sensitive information, to list a few. Therefore, a system is needed that detects whether a click on a link is intentional or unintentional and, if unintentional, can then prevent it. This paper proposes a micro-behavioral accidental click detection system (ACDS) to prevent slip-based human error. A within-subject-based experiment was conducted with 20 participants to test the potential of the proposed system. The results reveal the statistical significance between the two cases of intentional vs. unintentional clicks using a smartphone. Random tree, random forest, and support vector machine classifiers were used, exhibiting 82.6%, 87.2%, and 91.6% accuracy in detecting unintentional clicks, respectively.

## 1. Introduction

Over 95% of successful cyberattacks are the result of the weakest security chain link, human error, according to Cybint [1] and the IBM Cyber Security Intelligence Index Report [2]. Human error ‘is a generic term that involves all those instances where a planned activity fails to achieve its intended outcome’ [3]. Human error may result from inadequate security awareness, negligence, or health factors, including psychological or emotional effects, among other reasons [3]. These factors increase the likelihood of human error, especially if several factors align to cause the error.

However, even without most human error factors, a chance of unintentional error among even experienced individuals still exists. According to [4], unintentional human error, known as slip-based error according to [3], in the human error model, occurs on average one to three times per hour. If an error is related to installing malware or clicking on a phishing link, the effect can be catastrophic.

Phishing attacks have been developed to take advantage of slip-based errors for financial reasons. Slip-based human error occurs when a user commits a task unintentionally, resulting from an unintentional click, touch on a screen, or manipulated script forcing the user to click or touch a malicious link.

According to the IMC Group, since the start of COVID-19, the FBI reported an increase of 300% in cyberattacks and phishing attacks [5], all of which provide clickbait for users, taking advantage of the pandemic. Over 18 million phishing emails are sent per day, according to Business Insider [6]. According to Symantec [7], out of every 4200 emails, one email results in a successful phishing attack, and according to CSO Online [8], phishing attacks account for over 80% of reported incidents that cost over $17,500 per minute. Proofpoint reported that 88% of organizations were subject to phishing attacks in 2020 due to remote work forced by the pandemic [9]. Accidentally clicking on these phishing links regardless of the cause leads to human error consequences. Some are minor, and others are catastrophic, resulting in the loss of millions of dollars and public embarrassment.

As phishing attacks become more advanced and exploit slip-based human errors, it has become prominent for countermeasures to be put into place to safeguard organizational assets. Therefore, demand exists for a system that detects whether a click on a link is unintentional or intentional.

This paper proposes an accidental click detection system (ACDS), a micro-behavioral based ACDS, in which the system collects information from sensors presented on a smartphone before, during, and after a click to determine whether the click is unintentional or intentional and then proceed with or revert the action.

This paper presents the following contributions:

1. A proposed micro-behavioral measurement before, during, and after link clicks to prevent phishing attacks;
2. A system capable of detecting unintentional clicks on links by analyzing micro-behavior;
3. A classification model differentiating whether a click on a link is unintentional or intentional;
4. Validation of the capability of the proposed system.

The scope of this paper is detecting an unintentional click or touch on a link presented on a smartphone screen where the user knows that the click is an unintentional, slip-based error, yet the click is still made. The smartphone environment is chosen due to the vast capabilities of smartphones and because most phishing attacks result from unintentionally clicking on a phishing email on a smartphone. According to Wandera [10], 87% of successful phishing attacks occur on smartphones.

The remainder of this paper is organized as follows. The background and literature review on slip-based human error detection is presented in Section 2. The hypotheses and objectives are provided in Section 3. The ACDS design is revealed and described in Section 4. The methodology, experimental design and procedures, and data analysis are discussed in Section 5. The results are provided in Section 6. Finally, the conclusion and future work are presented in Section 7.

## 2. Background and Literature Review

### 2.1. Human Error

Human error has been studied for many decades in various fields, including psychology [3,11] and information security [12,13,14], and researchers continue to discover new aspects related to errors and suggest new solutions as technology advances. Some suggested solutions take advantage of psychological theories and discoveries to reduce, limit, detect, and prevent errors. Some are best practices and policies, and others are technical countermeasures. Together, they form a strong solution to the human error problem.

However, as human error may result from many possibilities due to the broad nature of errors, researchers target the human error problem according to Reason’s model of human error [3]. The model states that errors can be either unintentional or intentional, where unintentional human error can be a result of lapses of memory, related to forgetting to do something or how to do it (e.g., forgetting to close a port on the firewall) or slips of action related to not performing an intended action (e.g., unintentionally clicking on a phishing link). Both types are considered skill-based errors. According to Reason’s model, intentional errors can be rule- and knowledge-based mistakes, where a user acts intentionally but performs the action wrongly due to a lack of knowledge. Alternatively, the errors can be violations in which a user does not follow the guidelines or policies and acts without care or commits sabotage, a form of insider threat. Therefore, most research work has targeted intentional or unintentional human error to prevent it.

### 2.2. Intentional-Based Human Error

Various studies have been conducted to detect intentional-based human error, either knowledge-based error or violations. The authors in [15] proposed an intentional knowledge-based human error detection system by analyzing eye-gaze behavior while completing the sensitive task of configuring a firewall to determine whether a user is an expert at the task and possesses enough knowledge. The authors analyzed eye fixations, dwells, and saccades in an experimental setting and reported that experts exhibited less of each behavior while completing a task than inexpert users. The system reported 99.74% accuracy in detecting the human error likelihood using the KStar classifier, which could be a valuable solution to prevent human errors before they happen.

Furthermore, the authors in [16] proposed a human-computer interface (HCI) projected technique to identify knowledge-based human error resulting from low situational awareness in complex processes. The proposed user interface reconfiguration considers the projected future system state. If the current state is not what is projected, the interface alerts the user to correct the expected error. Unfortunately, no system results have been reported.

The authors in [17] took a different approach by designing a system that anticipates human error by collecting periodic information from questionnaires, which determines the knowledge level of the user at a specific time. The system analyses the collected data and predicts whether a human error is likely. However, no system results have been reported.

For violation-based human error, when a user intentionally performs a task (e.g., clicking on a phishing link knowing it will cause harm), a form of insider threat, the authors in [18] proposed monitoring the physiological signals of an individual while performing a task. They reported that a signal deviation happens once a user starts a violation, where the ECG and GSR rates increase, and the skin temperature decreases suddenly. The system reported a violation detection accuracy of 100% using the nearest neighbor classifier on an experiment with 15 subjects.

Furthermore, the authors in [19] proposed the intent-based access control system and patent [20], allowing the detection of the user’s intent, the execution probability, and the motivation for the malicious intent. The system reported an accuracy of 100% for intent detection by analyzing the user’s brain signals using an electroencephalogram (EEG) with visual stimuli.

The authors in [21] further proposed a novel system and patent [22] to examine the usage of head micromovement to detect malicious intent using visual stimuli and reported an accuracy of 100% intention detection with a lower accuracy (70%) in motivation detection compared with the analysis of EEG signals to detect a user’s motivation for an intended action.

The authors in [23] designed a system to detect intentional malicious driving by analyzing the driver’s behavior from data exported from an OBD-II sensor placed on the vehicle. They reported 99.95% accuracy using random tree and forest classifiers to distinguish between normal and malicious driving. Furthermore, violation-based human error has been researched with high detection accuracy using facial recognition [24] and baseline anomaly detection [25] based on predefined scenarios at 93% accuracy and using an artificial immune system [26] with 86.34% detection accuracy. Most intentional human error, whether knowledge-based or violations, can be detected with high accuracy and mitigated using previously designed and tested systems in conjunction with insider threat mechanisms and guidelines, including the Insider Threat Mitigation Guide by Cyber Security Infrastructure Security Agency released in 2020 [27].

### 2.3. Unintentional-Based Human Error

Some work has been conducted to detect unintentional human error, whether lapses or slips, from a technical point of view. Reason’s model states that lapses result from memory failure or omitting items, where a user says, “I forgot!” in response to an error, whereas slips are unintentional actions resulting from attention failure, distraction, or manipulation, where a user says, “I did not mean to do it” in response to an error. Slips include pressing the wrong key or clicking on a phishing link, to list a few.

Solutions to lapses include segregation of duties where two or more users must approve the action to be performed (e.g., configuring a firewall) and an automated checklist, where errors can be detected if the users fall out of sequence or forget a step in a process. Intelligent helper machines can also reduce lapse-based errors. Actions are monitored automatically and corrected if they are mistakes.

The authors in [28] investigated a lapse-based error by analyzing EEG signals during tactile human-machine interaction. The authors reported a change in EEG signals when a user forgets to perform an action, and the experiment analysis reports a 68.2% accuracy in differentiating between correct and error actions in 360 to 510 ms. As lapse-based human error may result from an illness (e.g., Alzheimer’s disease), among other related health issues, detecting lapses is challenging without multiple sensors placed on the user (e.g., EEG sensors). Therefore, most research has focused on the system expectations of a normal and correct procedure to detect a lapse-based error, which includes errors made by medical doctors, pilots, and astronauts. Moreover, over 60% of errors result from human error [29,30].

In slip-based human error, most intentional, knowledge-based errors or violations would not be detected. Even lapse-based human error solutions would not detect slips. The user can be a qualified expert who has performed an action thousands of times yet can still make an error. Attention detection systems may assist in detecting slip errors.

The authors in [31] studied a numerical entry human-based error with bank employees, using human behavior modeling, EEG analysis, data mining, and linear discriminant analysis (LDA). They detected the unintentional error with 67.84% accuracy using EEG data only, 64.42% accuracy using behavior modeling data only, and 74.84% using both EEG and behavior modeling data.

Furthermore, the authors in [32] designed a game for grasping and pushing a ball to detect whether a user intentionally or unintentionally missed the ball. They used acoustic phonetic data while playing the game and separated the prior and post actions of grasping and punching the ball. They input the data into C45 and ID3 algorithms and reported accuracy measures of 62% and 46%, respectively.

Because most unintentional-based human error involves a human movement, often hand movement, the authors in [33] investigated the ability to differentiate between intentional and unintentional hand movement, which can help detect unintentional clicks. The authors ran an experiment on seven individuals and collected EEG data. Then, they input the data into an optimized LDA algorithm using particle swarm optimization, achieving 86.4% accuracy in differentiating between intentional and unintentional hand movement.

The authors in [34] used eye movement and pupil features to detect unintentional human error resulting from mental workload while performing several tasks. They achieved 84.5% accuracy on average using the random forest classifier among the 25 participants in the experiment.

Furthermore, Google [35] developed three solutions to detect unintentional clicks on their ads on different platforms, and all seem to reduce unintentional ad clicks. The first solution is to introduce a delay before the ad can be clickable. The second is to disallow app icon clicks on ads, and the third is to revert any corner ad image clicks. Usually, when users are confident about their clicks/selections, the click is executed on or around the center of the image/link, not the edge or corner, which reduces unintentional ad clicks by 50%, according to Google [35]. Table 1 summarizes the human error per category and type and lists the method and accuracy for each detection. The detection accuracy dramatically declines when detecting unintentional human error, whether lapses or slips, compared to intentional human error, whether knowledge-based or violations.

Although related work demonstrates promising results in detecting slip-based and general human error, no previous work has provided a solution targeting slip-based errors on smartphones using micro-behavior movements before, during, and after the error, considering the error context. Therefore, due to the importance of detecting unintentional human error and detecting and preventing slip-based human error, especially in smartphones, this paper proposes a micro-behavioral-based unintentional ACDS projected to minimize the human error of an unintentional click on a phishing link on a smartphone. The next section provides the hypotheses and objectives for the design and evaluation of the proposed ACDS.

## 3. Hypotheses and Objectives

Attackers present sophisticated methods to gain access to system resources. Some are psychological to subconsciously deceive a user to unintentionally click on malicious links, a slip-based human error. Attackers take advantage of and sometimes cause attention failure, using distraction and manipulation to gain access. Thus, it is important to develop the following hypotheses to model unintentional vs intentional clicks or touches on a link on a smartphone to combat this threat because 87% of successful attacks target smartphones, which possess various sensors allowing human behavior modeling [36,37,38,39,40].

### 3.1. Hypotheses

Main hypothesis:

**Hypothesis** **1.** *Unintentional clicks can be detected and reversed by analyzing the micro-behavior of the user before, during, and after the click.*

The rationale behind this central hypothesis is derived from the human factor theory of unintentional causation [41,42], which states that slip-based human error can result from an inappropriate response or activity. The ability to detect unintentional hand movement, which reached over 87% accuracy when analyzing EEG signals [33], supports this hypothesis. Thus, EEG signals differ in intended vs unintended hand movement. While EEG signals related to unintentional hand movement differ, according to the main hypothesis, a different hand movement behavior measured at the micro-level could be sensed by the various equipped smartphone sensors.

Therefore, to support the main hypothesis, it is important to develop the following hypothesis:

**Hypothesis** **2.** *Hand micro-behavior before, during, and after a click exhibits a statistical difference between unintentional and intentional clicks.*

To investigate the ability to detect unintentional vs intentional clicks, it is necessary to investigate whether a statistical difference exists between unintentional and intentional clicks, and if so, this supports the creation of a behavioral model, supporting the central hypothesis.

### 3.2. Objectives

The developed quantitative objectives are as follows:

1. To design the ACDS where micro-behavior before, during, and after a click are collected and analyzed to determine whether to revert or continue with the operation.
2. To investigate the statistical difference between unintentional and intentional clicks.
3. To provide a model to classify whether the micro-behavior of a click is unintentional or intentional.
4. To evaluate the capability of the ACDS in detecting unintentional clicks in a real-life scenario.

## 4. Accidental Click Detection System Design

Smartphone sensors, including accelerometers, gyroscopes, magnetometers, gravity, and the screen, present numerous human behavior modeling and detection capabilities. This monitoring can cover walking patterns, mood, health (e.g., heart rate), and fall detection, among other possibilities. Therefore, the smartphone can provide data supporting the hypotheses and detect unintentional human error, such as unintentional clicks on phishing links, especially as most successful phishing attacks target smartphones. The unintentional ACDS design equipped with smartphone sensors is provided and described in this section.

The system comprises five main components:

1. Sensors: The ACDS relies on input from sensors to detect whether a click is unintentional or intentional. The selected sensors to evaluate the click are the screen, touch sensor (initiating the investigation), accelerometer, gyroscope, magnetometer, gravity sensor, linear acceleration sensor, rotation sensor, and pressure sensor.
2. Recording unit: All sensor data are recorded on a loop of 3 s to capture the before, during, and after sensor click data, including a screenshot of the clicked area, which serves as an additional verification of a possible unintentional click. The limit of 4 s was selected for analysis, as each click lasts for 40 ms to 500 ms, depending on the user behavior and situation/condition, and 1.5 s before and after the clicks are assessed.
3. The preprocessing unit includes three parts:
  - Screen and touch sensor data: A screenshot is taken to analyze what the user clicked on and the behavior of the click, including the time and start and end of each segment of the click, such as before, during, and after clicking;
  - General sensor data: All sensor data are prepared by segmenting the data into three segments: before, during, and after clicking;
  - Text extraction: The clicked area is prepared for optical character recognition to determine what a user clicked on and the general text around the clicked area.
4. Classification: Data are input into the classifiers to report whether a click is unintentional or intentional. Two classifiers are trained: (1) classifying the touch sensor data and general sensor data and (2) classifying the text in the text matching settings, where the extracted text from the click is mapped against the actual link and tone, analyzing where the tone of the text is present on the page to determine whether the text aims to convince a user to click on a link.
5. Decision-maker: The system decides to revert the clicked action and present an alert or allow the action. The decision-maker considers all classification results to make the decision. Details on the decision-maker are provided in the Data Analysis section.

**Figure 1:**
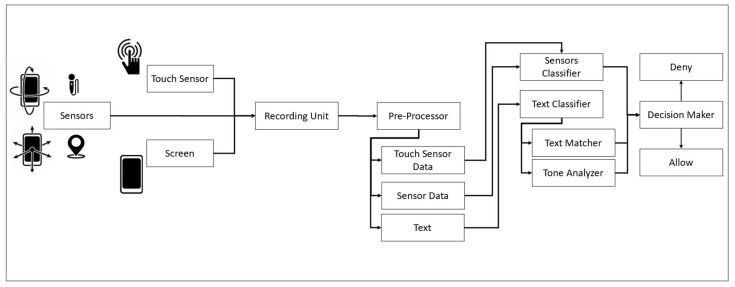
Accidental click detection system (ACDS) design.

The five components of the ACDS allow for detecting whether a click is unintentional or intentional and deciding whether action should be reverted or allowed. Figure 1 depicts the ACDS design.

## 5. Methodology, Experimental Design, and Data Analysis

### 5.1. Methodology

Human error data can be collected by observation, error reporting, or systemized data collection [43]. The best option in this research context is systemized data collection, especially in determining human micro-behavior as a possible metric in reaction to unintentional vs intentional clicks. Therefore, the methodology employs human experimentation while collecting micro-movements from smartphone sensors. The following sections provide details on the experimental design, procedure, and discussions to provide a training dataset. Random tree, random forest, and support vector machine (SVM) classifiers were analyzed using the decision-making component of the ACDS to test and validate whether the findings support the hypotheses.

### 5.2. Experiment

The experiment has a within-subject design where each participant faces scenarios that lead to unintentional and intentional clicks. The sensor data were collected, and the micro-behaviors were recorded for analysis by the ACDS. Future research directions may also be highlighted.

#### 5.2.1. Experiment Goal

The experiment goal is to provide a dataset, dataset 1, of human micro-behavior in the settings of unintentional and intentional clicks or touches on a smartphone. The dataset was trained using the random tree, random forest, and SVM classifiers in the three components of the preprocessing phase: touch sensor data, sensor data, and text. Then, a real-life dataset, dataset 2, of other participants was generated while making unintentional and intentional clicks or touches. This second dataset was used to test the capabilities of the ACDS in differentiating between the two cases and report the system accuracy. The first dataset was used to train the ACDS to learn how to differentiate between the two cases, and the second dataset was used to evaluate the ACDS.

#### 5.2.2. Subjects

In a controlled setting, 20 male and female participants between 19 and 45 years old participated in the experiment. No participant had an essential tremor illness, leading to involuntary rhythmical shaking, and all participants were right-handed. The participants were recruited using flyers and emails to participate in the experiment. The flyer/email stated that participants are needed to play a game on a smartphone. Participants who finish the game with the highest score were awarded a gift card. The rationale behind the prize was to influence participants to play with the closest attention, as attention plays a significant role in slip-based human error.

#### 5.2.3. Procedure

Participants started by signing a consent form detailing that they would be playing a game of attention, where all behavioral data would be collected for analysis. After signing the consent form, participants were individually provided with a Samsung Note 5 (Tabuk, Saudi Arabia) with the game app installed and launched. Participants were requested to click on start to start the game, starting the data recording and creating the first dataset for training the classification algorithm.

The recorded data include the following:

1. Accelerometer, units: m/s^2^,
2. Gyroscope, units: rad/s,
3. Magnetometer, units: μT,
4. Gravity, units: m/s^2^,
5. Linear acceleration, units: m/s^2^,
6. Rotation sensor, units: quaternion,
7. Time, units: ms,
8. Touch sensor, and
9. Screen.

Each sensor was set at 100 samples/s, which was the maximum possible using a Samsung Note 5. All x, y, and z coordinates were captured.

#### 5.2.4. Game Design

**Figure 2:**
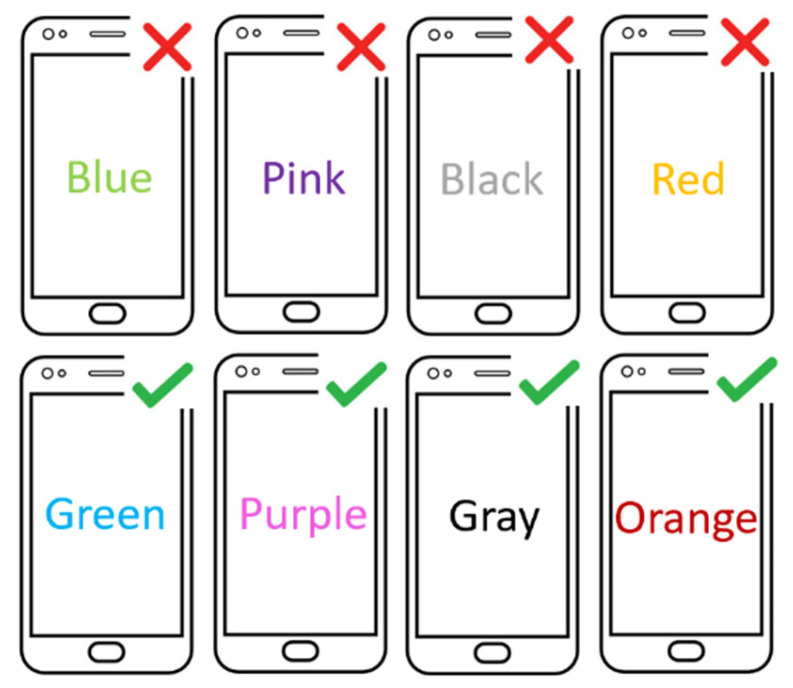
Example of correct and wrong cases.

The game was designed as follows. A participant must click on the text for a color if the correct color is presented but not the color name/word and must click on the white space below the text of the color if the color is wrong. The selected correct colors are blue, pink, black, and red but not any other colors, including green, purple, grey, or orange. Each word is written in a different color, sometimes similar and sometimes different from a color, to cause the possibility of unintentional clicks. Figure 2 depicts an example of the correct and wrong choices used in the game. Each color name is centered on the screen for easy and consistent reach. The game follows the Stroop effect [44,45,46], a neuropsychological test that challenges the brain with two different kinds of stimuli: color and name of the color, serving the need to induce unintentional clicks.

Each session includes trials and confirmations following each trial. Each trial lasts for more than 3 s per trial, but less than 4 s. The participant must click during this 1.5 s on the screen. If a click is not performed, the trial is discarded, and a new trial is initiated. After a click on the screen, when the fingertip is lifted, a delay of 1.5 s is triggered to capture the post effect. Each participant completed 60 trials. After each trial, participants were asked to click on the screen and choose whether their selection was thought to be correct, wrong, or unsure for verification purposes, which was the confirmation.

**Figure 3:**
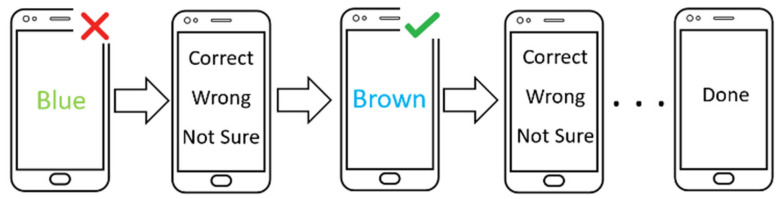
Flow design of the game.

The confirmation also serves as an intentional click class for the dataset because each click is an intended click, providing 60 confirmations. The duration for the confirmation step after each trial was set with no time limit and served as a necessary gap between trials to ensure each collected data point is related to the trial and that no overlap can occur. Therefore, each session lasted 180 s plus the time taken for each click and the time taken in each confirmation step. Figure 3 depicts the flow of the experiment.

Out of the 60 possible selections, 50% were marked as wrong selections if they were made, and 50% were marked as correct selections if they were made. The rationale behind the 60 selections, each in 3 s or more, is to have enough data in the dataset for analysis. The rationale behind the 3 s or more per trial is to invoke a fast response that a participant may get right intentionally or wrong unintentionally, similar to the experiment done by [47], where a participant was stimulated and requested to move the mouse right or left in a specific time to capture the mouse dynamics. In this experiment, all participants used their right hand in two settings:

1. Holding the smartphone and making selections with the right hand;
2. Holding the smartphone with the left hand and making selections with the right hand.

The rationale behind the two settings is to investigate the influence of the usage style on the ACDS. To avoid the influence of one setting following the other, 10 participants started with the first setting followed by the second setting, and the other 10 participants started with the second setting followed by the first setting. The rationale behind giving two groups of participants different starting settings is to remove the influence of the trial of the first setting on the trial of the second setting, known as the matrix box for a factorial design experiment [48].

Therefore, it is concluded with 10 participants in both the first and second settings to evaluate whether any influence on the ACDS occurred and a dataset of 20 participants making several unintentional and intentional clicks per session for two sessions was obtained, providing 2400 trials and 2400 intentional clicks to confirm the results after each session. There was no guarantee that an unintentional click would be made from the first 2400 clicks; thus, each selection was followed by the user stating whether it was selected by mistake to determine an unintentional click. Each correct click followed by a confirmation of a correct click was considered an intentional click. All other clicks were omitted, as one would not know whether they were made by mistake. An additional trial was added so that each participant completed 60 trials per session to compensate for clicks not made.

A difference exists between the number of correctly clicked colors and the 2400 intentional clicks in the confirmation section related to the time factor, attention level, and stress level. The ACDS uses the difference to differentiate between the two intentional groups, i.e., confirmed intentional clicks and hesitant intentional clicks. The selection a user makes is recorded in the database and marks the number of correct vs wrong selections and whether the confirmation step after each trial matches the correct result of selection. The total wrong and correct selections are reported in the analysis section, as they serve as the training dataset for the classifier.

#### 5.2.5. Form Design

**Figure 4:**
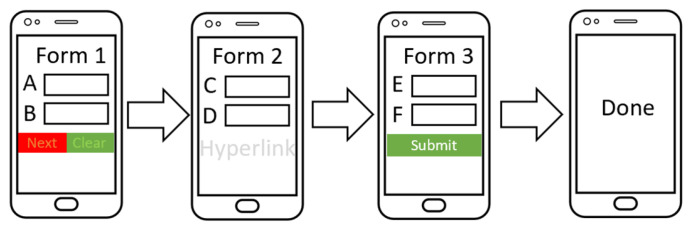
Flow design of the form.

The second dataset was created to use as a new untrained dataset to validate the capability of the ACDS by requesting participants who finished playing the game to complete an electronic form on the smartphone. The form also allowed the investigator to contact the participant with the highest game score. Participants entered two data types in each section on the three-section form and clicked next to move to the second section. The data include name, gender, date of birth, phone number, a short biography, and email. Participants must click next to move to the next page and click submit on the last page to submit the data. However, the next button was red and on the left, whereas the clear button was green and on the right in the first form section. This setup is the opposite of the typical design and may cause an unintentional error that would clear the data from the form. The second form section after completion shows no buttons. Once the users enter their date of birth and phone number, they are expected to click on the white space under the fields. A hyperlink in white was placed there, which would open a new page if clicked, simulating an error. Then, the next button appears under the form. Finally, after the participants entered their biographies and emails, they could click on the submit button to submit the data. The first two unintentional forms were designed to cause a slip-based error, and the final form recorded an intentional click. The 20 participants completed the form with three sections, comprising 40 possible unintentional clicks and 20 intentional clicks. A total of 10 participants used one hand, while another 10 participants used two hands. Figure 4 depicts the three-section form design. Table 2 provides a summary of the two datasets.

### 5.3. Data Analysis

After collecting the data from the 20 participants for Dataset 1 on the game for training the ACDS and for Dataset 2 on the form for evaluating the ACDS in both settings (one or both hands), Dataset 1 comprised 240 trials and Dataset 2 comprised 60 trials. The data were converted to CSV format for analysis following the ACDS preprocessing component. The dataset comprises all sensor data, a screenshot of where a click was made, and a timestamp in milliseconds. Each trial had 3 s or more of recording: 1.5 s before the start of the click, 1.5 s after the click, and a few milliseconds were recorded while the click was made, which is the duration of the touch on the screen.

The first step is labeling the data in Datasets 1 and 2 for unintentional and intentional clicks. The labeling in Dataset 1 is to train the ACDS, and the labeling in Dataset 2 is to evaluate the ACDS. Each confirmation (correct, wrong, or unsure) in Dataset 1 was extracted and mapped to the stream of trials to achieve this step, where the color was correct, and a user clicked on it or on wrong or clicked under the color name on the empty space. If the user clicked correctly while making the correct click, the trial was marked as a successful intended click. If the user clicked on wrong while making a wrong click, the trial was marked as a successful unintentional click. The analysis indicates that, out of the 240 trials, 89 were confirmed unintentional clicks, 42 with one hand and 47 with two hands, and 104 were confirmed intentional clicks, 61 with one hand and 43 with two hands. The remaining trials were removed, such as when a user stated a correct click while making a wrong click, stated a wrong click while making a correct click, or stated that they were unsure. Dataset 1 also includes 120 trials of the confirmation of intentional clicks. Table 3 summarizes the successfully collected data of unintentional vs intentional clicks for Dataset 1, and Table 4 summarizes the collected data on intentional clicks in the confirmation vs. during gameplay.

**Figure 5:**
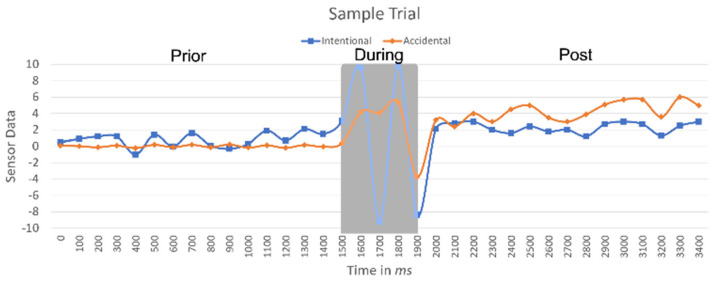
Illustration of a sample trial.

Dataset 2 has 60 trials, 40 for unintentional clicks and 20 for intentional clicks after submitting the form. All trials were determined to be valid, with no noise in the data, to evaluate the ACDS. When making the intentional or unintentional click, each trial was extracted following the exact method applied in Dataset 1. Each trial was for 3 s or more, including 1.5 s before the click, 1.5 s after the click, and the milliseconds during the touch on the screen, taking advantage of the touch sensor to locate each trial in both datasets. Figure 5 depicts an illustration of a sample trial.

After constructing the datasets and labeling each trial, intentional vs unintentional in both datasets and intentional in the conformation vs gameplay, further data preprocessing was performed on the touch and sensor data, touch duration, accelerometer, gyroscope, magnetometer, gravity, linear acceleration, rotation sensor, and pressure sensor. Each sensor data point in the trial was divided into three, before, during, and after a click, providing 24 features for training the ACDS classifiers plus the standard deviation calculated per segment per participant (25 features).

However, before training the classifiers, data were normalized and smoothed. Then, the principal component analysis was applied for dimensionality reduction. Next, feature selection was performed using wrapper subset selection using the random tree, random forest, and SVM classifiers to select the best features per classifier. Three classification models were created for the intentional vs unintentional trials and three classification models for the intentional trials in the conformation vs gameplay for the one-hand and two-hand settings, a total of 12 classifiers. The following section reports the classifier accuracy.

Furthermore, for the recorded images, the screenshots of the center of the click were analyzed. Optical character recognition was applied to extract the text on a page. Two methods were used on the extracted text, matching the text with the link and extracting the text tone on the screen. If a text contained a link, the link was matched to the extracted text. If the text and link matched or did not match, a trust score was assigned from 0 to 1, depending on the link type, such as short links, known phishing websites, and so on. For the tone analysis of the text on the screen, the IBM Tone Analyser API [49] and Geoflx [50], a chrome extension that detects text sentiment on a web page, were used to extract the tone. If the tone was negative or positive, a trust score was assigned from 0 to 1, depending on the tone strength.

**Figure 6:**
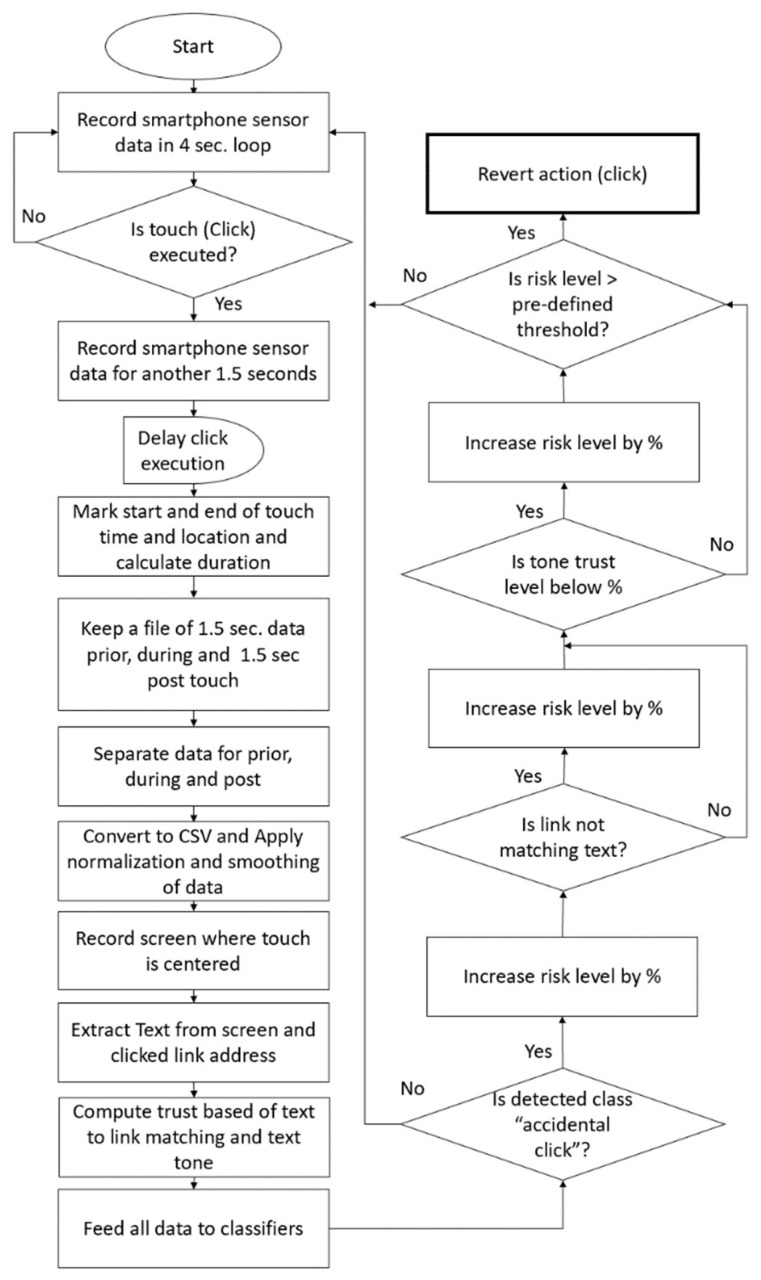
ACDS data analysis algorithm.

Finally, the decision-making component decides whether a click was unintentional or intentional based on the classification results, text matching, and text tone results. The decision-maker assigns a weight value for each of the three components and can be tweaked as needed. Text analysis was only performed on Dataset 2 to possibly improve the ACDS. Figure 6 depicts the ACDS algorithm for detecting unintentional vs intentional clicks, and the next section provides the ACDS results.

## 6. Results

### 6.1. ACDS Results

Objectives 2, 3, and 4 are achieved in this section, and the hypotheses are tested. The classification results are reported in each condition (one hand and two hands) to classify unintentional vs intentional clicks and intentional clicks in confirmation vs gameplay and report on the capability of the ACDS.

First, to achieve Objective 2 and to investigate the statistical difference between unintentional and intentional clicks, a *t*-test was applied before a click during unintentional vs intentional clicks. The signal gradually increases in fluctuation in intentional-based clicks while remaining almost flat in unintentional-based clicks, *p* < 0.042. Furthermore, a t-test was applied during the unintentional vs intentional clicks, finding *p* < 0.001 when the signal deviates significantly for a brief period in intentional-based clicks while fluctuating at lower power for a longer period during unintentional-based clicks. Finally, a *t*-test was applied after a click for the unintentional vs intentional clicks, finding that *p* < 0.02 when the signal returns to the prior click signal fluctuation behavior, whereas the signal continues to fluctuate at high power in unintentional-based clicks compared to the prior stage. The results support the hypothesis that hand micro-behavior before, during, and after a click exhibit a statistical difference between unintentional and intentional clicks. The statistical difference exhibited similar results in both scenarios (one-hand and two-hand settings). In the two-hand setting, the fluctuations in the during and post stages were significantly higher: *p* < 0.01 in the prior stage, *p* < 0.001 in during stage, and *p* < 0.007 in the post stage.

Twelve classifiers were created when training the ACDS: six in the one-hand setting and six in the two-hand setting. The six classifiers in each setting were divided between unintentional and intentional-based clicks and intentional clicks in confirmation vs gameplay. The three classifiers per condition were the random tree, random forest, and SVM classifiers. Table 5 lists the classification results of the 12 classifiers. All were trained by Dataset 1 while applying 10-fold cross-validation.

The results reveal that the SVM was the best classifier for detecting unintentional vs intentional clicks, achieving 95.1% in the one-hand setting and 97.2% in the two-hand setting. This result achieves the third objective to provide a classification model for whether the micro-behavior of a click is unintentional or intentional. The results indicate low but promising accuracy when differentiating between intentional clicks in the confirmation page and intentional clicks in the gameplay. The best classification accuracy reached 76.6% using the SVM classifier.

The classification models were applied on Dataset 2 while completing a form to evaluate the capability of the ACDS. In addition, 20 unintentional clicks and 10 intentional clicks were made, once using one hand and once using two hands. The classification results are presented in Table 6.

The results demonstrate that the SVM classifier performs best in detecting unintentional vs intentional clicks, with 86.5% in the one-hand setting and 91.6% in the two-hand setting. The results achieve the last objective, to evaluate the capability of the ACDS in detecting unintentional clicks in a real-life scenario and support the main hypothesis that unintentional clicks can be detected and reversed by analyzing the micro-behavior of the user’s response before, during, and after the click.

For Dataset 2, both link matching and tone analysis reported high-risk results with a trust level close to zero, as in the first form, the text cancel was green, and in the second form, there was no text but a white background hyperlink. Opening a new web page on an empty screen after clicking on white space or the cancel button is a phishing approach that the system was manually trained to detect. Each condition can be selected to either block the action, revert, and show an alert or continue the operation.

### 6.2. Discussion

To better evaluate the proposed ACDS system, two datasets were constructed, one for training and testing the classifier for the two conditions, i.e., intentional vs. accidental clicks, and one for evaluating the classifiers on a different dataset that the classifiers never learned from, a more realistic approach for verifying the accuracy of the classifiers. The classification accuracy verification used was 10-fold cross validation as a validation approach instead of separating the dataset to 60% training and 40% testing, as cross validation provides the average of training and testing various dataset scenarios and report the average results, which is a less biased approach. The testing of the classifiers on newly generated dataset should result in less classification accuracy; however, the resulted accuracy results are less biased and more realistic when compared to training and testing on the same dataset.

However, further dataset creation is desired to reduce bias and return a comprehensive accuracy reporting, that is by increasing the population in the experiment, with different demographics, and using different smartphones equipped with different sensors or sensors manufacturers to conclude the optimal classification accuracy reporting.

The ACDS system when compared to the literature shows higher acceptability, accessibility, and accuracy. Even though using EEG signals and eye movement show the ability to detect slip-based human error with an accuracy of 86.4% [33] and 84.5% [34], respectively, the applications may be limited, as they require either special equipment to be placed on one’s scalp to capture the EEG signals that are susceptible to noise given the low amplitude EEG signals have, measured in the micro volt, or require a live stream capture from a camera at all times. Therefore, utilizing the sensors that a typical smartphone already has in order to detect slip-based human error may be a better accepted solution to the general public. Micro-behavior analysis provides more acceptability and accessibility to the user and require no additional sensors to provide a slip-based human error detection system especially in day-to-day smartphone usage. The ACDS also reports a higher accuracy when compared to existing slip-based human error detection of an 86.5% using one hand and 91.6% when using two hands an increase in accuracy of ~+0.1%–+5.2% when compared to EEG-based solution and ~+2%–+7.1% when compared to an eye movement-based solution.

### 6.3. Limitations

The ACDS system shows some limitations that relate to delay and power consumption. The duration for data collection and analysis to obtain classification results after a click was 5 s, which is a delay the user must accept until the analysis speed can be increased, as smartphone technology continues to improve. The system can provide an alert after a click if the system detects an unintentional click and can present an alert that a link address will open so that the user can choose to continue or revert the action.

The current system consumes 5.5 mA/s, excluding classification and screen power consumption; therefore, it should not be running all the time. Other metrics where a system starts or stops the recording can be proposed in the future to optimize the system and benefit from its capabilities.

The acceptability of the ACDS has not been fully evaluated, but it is expected that the acceptability would be low in day-to-day life unless the system activates only when opening a chatting application or email because most phishing attackers use email or chat options.

One drawback of the system is detecting whether a click is aimed to open a new link or type in a box. All clicks were manually omitted to fill in the form; however, a metric to detect whether a new page would open is required for the ACDS to function better.

The present results reveal the potential of the ACDS in detecting unintentional clicks and differentiating them from intentional clicks; however, the current study has not been tested in day-to-day life. Thus, some false positives or negatives are expected. The system can be improved to learn from those mistakes over time to reduce false positives and negatives. Furthermore, the system may take advantage of user behavior modeling to detect intentional vs unintentional clicks, as they may vary by the user or by the user’s current condition, such as walking or running.

If a user does not know that they made an unintentional click, the ACDS will not be able to detect it as it heavily relies on the user’s behavior where a user notices that they clicked by mistake or a function on the screen appears that was not expected. It is said that no one can detect a liar if the one telling the lie does not know they are lying. This same applies to slip-based human error. If the user does not know they are making an accidental click, no behavior response will happen and the ACDS system will not be able to detect the accidental click. Usually, a user realizes they are making the accidental error prior, during, and mostly after the error is made, which is why the ACDS system needs to be able to detect the accidental click after the click was made.

One major limitation of the ACDS system is the delay in analysis, especially post-click, when it lasts 1.5 s at least. This is a tradeoff between accuracy/security and convenience, where if the post click analysis is removed, the acceptability and convenance improves but the classification accuracy degreases suddenly by 17.1–21.82% from the three tested classifiers. One possible solution is to consider the context of the click as if the click was on a website the user never visited before or from an email the user does not know. This may trigger the need to analyzing the post click or not, which may assist in reducing the delay.

## 7. Conclusions and Future Work

Human error takes different shapes and has multiple causes, which are abused by attackers to gain access and launch successful attacks. One of the human error types is a slip-based human error, in which a user makes a mistake because of insufficient attention, distraction, or manipulation. Phishing attacks, a form of deceiving the user to click on a link or download an infected attachment, have increased in number over the past years.

Phishing attacks use psychological concepts in the text to influence users to click on a link or download an attachment. Some are time-triggered based on specific events or expectations, and others are random to gain as much access as possible. Other phishing attacks force users to click on a link by having the whole page as a clickable link, by following the curser, by substituting an intended button location, or even by changing the text on the intended button to deceive the user. Most cases are detectable by the user before, during, or sometimes after the click is made, where the expected reaction does not meet the intentions.

Furthermore, smartphone use has increased, and employees rely heavily on the computational power they provide to perform day-to-day tasks. As smartphones possess sensitive information, a successful phishing attack may provide access to a company’s sensitive information, such as passwords or trade secrets, to list a few. An unintentional click is not necessarily just on links but can also be when pushing a code to production, sending a message, confirming a transaction, or a bank transfer. An unintentional click may lead to any of these cases. Because of the ease of use of smartphones and the sophistication of phishing attacks, an unintentional click or touch on a phishing link may lead to a successful attack.

Therefore, an ACDS was designed that takes advantage of the capabilities of smartphones (where most phishing attacks occur) to model unintentional vs intentional clicks. The system was trained based on data collected in a controlled experiment and evaluated the system using a real-life scenario of completing a form. The results support the capabilities of the proposed system, reaching 82.6%, 87.2%, and 91.6% accuracy using the random tree, random forest, and SVM, respectively. Furthermore, link matching and tone analysis of the text presented on a screen improved the system. The system assigns a risk factor if any link does not match the text a user clicked on or if the text tone on the screen is classified as influencing the user to click on a link, all of which are phishing strategies.

Future work may improve the system using various other classification algorithms, including deep learning to gain better accuracy results and test the system in real-life scenarios for a longer period where users report whether a click was unintentional or intentional. In addition, developing a model in which a user is expected to make an unintentional click to activate the ACDS beforehand to detect whether it occurs may improve the user experience, as the current system consumes power and is not user friendly due to the 5-s wait for every click. Furthermore, applying the regret theory [51], which states that regret is a behavioral response to an error, may improve the ACDS by detecting regret if a click is made.

Finally, studying human error probability, which is the number of errors divided by the number of opportunities for an error to occur, may assist in predicting an error. Other factors that cause an error can also be considered, such as detecting that a user has low attention or is being manipulated or distracted, to improve the ACDS.

## Figures and Tables

**Table 1:** Human error categories and detection accuracy.

Error Category	Error Type	Method	Accuracy	Ref.
Intentional	Knowledge-based	Eye gaze using KStar	99.74%	[15]
		Human Computer Interface	N/A	[16]
		Questionnaire	N/A	[17]
	Violations	ECG, GSR, and temp. using nearest neighbor	100%	[18]
		EEG using SVM	100%	[19,20]
		OBD-II using		[21,22]
		head micromovement using	100%	[23]
		OBD-II using random forest	99.95%	[28]
Unintentional	Lapses	EEG-based	68.2%	[28]
	Slips	Theoretical approach	N/A	[29,30]
		EEG-based using LDA	86.4%	[33]
		Eye movement using random forest	84.5%	[34]

**Table 2:** Training and evaluation datasets.

Dataset	Dataset 1	Dataset 2
Goal	Training	Evaluation
Method	game	forms
Number of clicks	2400	40
Number of Intentional clicks	2400	20
Duration	180 s + click duration and confirmation	Open
Trials	120 trials per participant + 120 confirmations per user	60

**Table 3:** Collected data on unintentional vs intentional clicks for Dataset 1.

Condition	Dataset 1	Dataset 2
1 hand intentional	42	20
1 hand unintentional	61	10
2 hands intentional	47	20
2 hands unintentional	43	10
Total trials	193	60
Total samples	19,300	6000

**Table 4:** Collected data on intentional clicks in the confirmation vs the game for Dataset 1.

Dataset	Dataset 1
1 hand intentional confirmation	60
1 hand intentional game	42
2 hands intentional confirmation	60
2 hands intentional game	47
Total trials	209
Total samples	20,900

**Table 5:** Classification results on Dataset 1 for training.

Dataset	Random Tree		Random Forest		SVM	
Hand settings	1	2	1	2	1	2
Intent vs. unintended clicks	89.7%	91.6%	92.3%	91.6%	92.3%	92.2%
Intent in confirmation vs. gameplay	57.1%	59.4%	63.3%	59.4%	63.3%	76.6%

**Table 6:** Classification results on Dataset 2 for evaluation.

Dataset	Random Tree		Random Forest		SVM	
Hand settings	1	2	1	2	1	2
Intent vs. unintended clicks	80.1%	82.6%	82.1%	82.6%	86.5%	91.6%

## Data Availability

The data presented in this study are available on request from the corresponding author.
